# Ten quick tips for developing a reproducible Shiny application

**DOI:** 10.1371/journal.pcbi.1013551

**Published:** 2025-10-13

**Authors:** Julien Brun, Greg Janée, Renata G. Curty

**Affiliations:** Research Data Services, Library, University of California, Santa Barbara, Santa Barbara, California, United States of America; Montreal, CANADA

## Introduction

**Fig 1:**
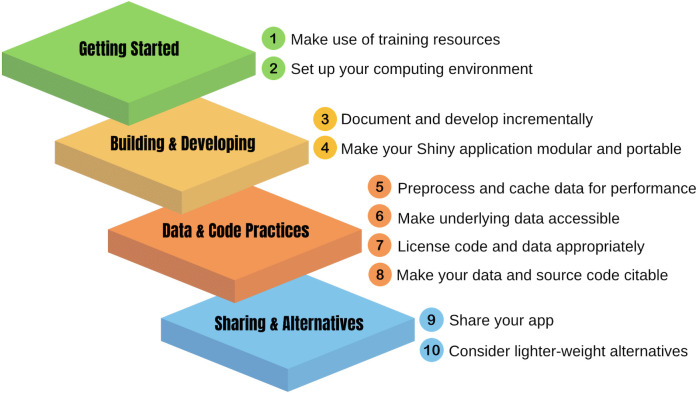
Overview of the 10 quick tips for developing a reproducible Shiny application. The tips have been organized into four main categories: getting started, building, and developing your Shiny app, data and code management best practices, and sharing your interactive data visualization as a full web application or using lightweight alternatives.

Shiny [1] offers a robust framework for making complex data accessible to broad audiences through interactive web applications. It is a valuable addition to the ecosystem of open source tools for scientific data analysis and visualization. It was first introduced as a package for the R programming language and, more recently, extended to Python. While it is a relatively modest learning curve to develop Shiny applications (hereafter “apps”) for a researcher knowing R or Python, it can be more challenging to maintain them over time. It is, therefore, important to take some extra steps to make the code and data behind apps accessible and inspectable by others. Here are 10 quick tips for enhancing the sustainability and reproducibility of your Shiny app. Fig 1 summarizes our 10 quick tips and how they can be organized according to four main categories: getting started, building and developing your app, data and code best practices, and sharing your Shiny app. Note that these recommendations focus on the open-source products of the Shiny ecosystem and not the more integrated publishing tools provided by licensed products. Although these recommendations are primarily focused on the R version of Shiny, these tips will apply to the Python-based Shiny ecosystem as well, with some minor adaptations. We also developed a companion Shiny app in R (https://github.com/UCSB-Library-Research-Data-Services/shiny-qt-example) to encapsulate of these tips into one application. This app repurposes the Old Faithful geyser application example that is used as a feature example of Shiny.

### 1. Make use of training resources

Coding Shiny apps is no different from writing R (or Python) code. However, some specificities of this framework will present a learning curve. One of these is the core concept of “reactivity,” which is more nuanced than it may appear at first. Although it is possible to develop Shiny apps with only a few components without an in-depth understanding of “reactivity”, it will be necessary to gain a better understanding of it before designing more complex apps [2]. Additionally, Shiny was developed with the intention that apps would be written following a certain structure, layout, and coding style. Getting familiar with the key concepts and looking at a few examples before creating your first app is thus recommended. This will ensure your app’s code is more predictable and easily understood by future readers.

The good news is that there are a lot of free resources online. To get started, we recommend:

- A. Exploring the Shiny Gallery [3] for a wide variety of examples with accompanying code,
- B. Checking online tutorials for numerous development tips and potential pitfalls to watch out for [4], and
- C. Delving into the definitive Mastering Shiny book [2], which is comprehensive, shows how to develop more complex and capable apps, and best of all, is freely available.

### 2. Set up your computing environment

Before you develop your app, updating to the latest versions of R and R packages will ensure you start with the best future-proof setup. If you do not make this a standard practice, you may find that your packages are years old. We also recommend using **renv** [5] to automatically capture and manage your computing environment, which will enhance the portability and durability of your app. Enabling **renv** can be done by checking a box when you start your Shiny project using the RStudio project wizard [6] or running renv::init() in any existing project.

We also recommend using a version control system, such as git, and a collaboration platform, such as GitHub, to manage your code. **renv** and git work together: by committing renv’s renv.lock, .Rprofile, renv/settings.json, and renv/activate.R files to your project repository, others should be able to rebuild the same computing environment you are using in your app.

Understand that **renv,** like other virtual environments, has limitations, especially from the perspective of long-term preservation, since it fails to capture low-level aspects of your computing environment, such as versions of the system-level libraries (e.g., Basic Linear Algebra Subprograms implementation such as the openBLAS library [7]) that R relies on. Reporting information about those system-level libraries may be significant for reproducibility in the long term. We thus recommend using sessionInfo(), in addition to **renv**, to record this information in a text file and adding it to your project repository. When developing a Shiny app in Python, **venv** can similarly be used to create virtual environments with the same limitations as mentioned above.

**Fig 2:**
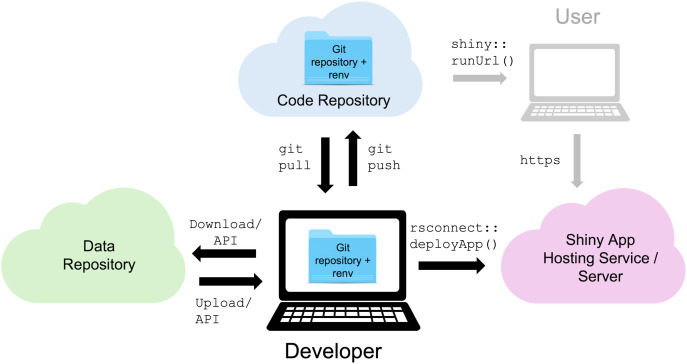
Overview of the recommended setup to develop reproducible Shiny applications: Use a version control system, such as git, and a code repository (e.g., GitHub and GitLab) to manage, archive, and share your code. Archive a copy of your data in a data repository (e.g., Zenodo, DRYAD, and DataONE) and mint a Digital Object Identifier (DOI) so you can cite it. Use **renv** to capture the computing environment used to develop your app and add it to your versioning system. Finally, publish your app on a Shiny server or shinyapp.io so users can access your app via the World Wide Web. Do not forget to add the links to the code and data repositories to your Shiny app landing page so users can access those resources.

Fig 2 summarizes this recommended setup to ease the long-term maintenance of your Shiny app and make it more open, trustworthy, and reproducible for others to use and build upon.

### 3. Document and develop incrementally

Shiny apps can get unwieldy very quickly due to the nested code structure of the UI components and the reliance on functions to enable reactivity. Some strategies regarding documentation and development will help you stay on track and save valuable time in the long run.

The usual dictum about code documentation applies to Shiny apps: document your code *now*, while you are developing it, and it is fresh in your mind because you will not find time to do it later. Furthermore, it is far easier and less daunting to build up documentation incrementally. A documentation consideration unique to Shiny is the handling of deeply nested functions, which occur in the user interface (UI) part of your app. Be sure to leverage the automatic indentation provided by IDEs such as Visual Studio Code and RStudio, and/or the “rainbow parentheses” option in RStudio (Tools -> Global Options -> Code -> Display). Regardless of the IDE you are using, it is often recommended to add comments at the beginning and end of each UI element [3]; see code in S1 Text for an example.

Regarding development, Shiny apps can be challenging to debug because your code is run by a separate server process that can be difficult to observe. It is highly recommended that you develop your app incrementally, by implementing only small changes at a time and testing your code after each change. Also, the debugging and tracing techniques described in Debugging Shiny applications [6] will be very helpful in understanding what is happening inside your app.

### 4. Make your Shiny application modular and portable

The organization of your files can impact how portable and maintainable your app is.

- Store the user interface and the R analytical code in separate files (in ui.R and server.R, respectively) to keep your code organized, as Shiny app in one script (app.R) can quickly get unwieldy as the complexity of your app grows. Store custom functions - code that performs data preprocessing and wrangling, and general declarations like ggplot themes - in global.R to keep the code in server.R strictly focused on your app’s logic.
- Load all the packages your app requires in global.R
- Organize the files for your app as follows:
  - Scripts (ui.R, etc.) go at the top level of your project folder
  - Store local data in a data subfolder
  - Store images (other than the plots your code produces), media files, CSS styles, and other content to be rendered in a www subfolder
- Within your app code, always use relative file paths. For convenience and portability across operating systems, consider using the file.path() R function and/or the **here** package to construct paths.

### 5. Preprocess and cache data for performance

Interactive applications require timely responsiveness to deliver an acceptable user experience. This means that in many, if not most, cases your Shiny app will need to operate on a processed version of your raw data that has been transformed for visualization and/or summarization. If your data is not integrated into your Shiny app due to size limitation, drive access, or other restrictions, it may be necessary to add a piece of code, preferably in global.R, that loads the data into a local file. This avoids downloading the data every time your app starts. S2 Text shows an example of storing a CSV file locally; R’s load() and save() functions can similarly be used to cache arbitrary R data structures and variables.

The same approach can be used to cache the outputs of data preprocessing. If the raw data needs some cleaning or summarization, and that processing is performed within the Shiny app, we recommend locally caching the output of such processing so it does not need to be run every time the app launches. Additionally, important strategies for optimizing Shiny apps’ performance are described in Chapter 23 of Mastering Shiny [2].

### 6. Make underlying data accessible

A Shiny application typically consists of both data and code that provides interactive access to that data. However, this interaction cannot replace the fundamental value of providing direct access to the underlying data, which is essential for open and reproducible science.

- If your Shiny app provides access to a dataset that has already been published and documented somewhere else (say, in a data repository), then cite that dataset, preferably with a DOI or other persistent identifier, both within the code and in the interface.
- If the data is available solely through the Shiny app, add a data download feature to the interface and provide documentation, including a recommended citation on the download page.

If the data is smaller than 100 MB, it can be convenient to add it to your code repository. However, code repositories, such as GitHub, generally do not prioritize long-term preservation to the same extent as data repositories. Therefore, we recommend archiving a copy of the dataset in a dedicated data repository accompanied by comprehensive documentation, including links to your other Shiny project components. Additional ethical assessments and considerations should be undertaken concerning the handling of sensitive data, including the prior removal of direct and indirect identifiers, which may negatively impact vulnerable communities, individuals’ privacy, endangered species or protected lands, leading to unintended harm [8].

### 7. License code and data appropriately

It is important to provide licensing information along with your app as it will let users know what they can legally and ethically do with your code. Most Shiny developers use some form of source code license for their apps. So-called “open source” licenses are recommended so that others can reuse and build on your work. A permissive license, such as the MIT license (see https://choosealicense.com for more options), is often sufficient, as Shiny app code is not intended to embed code requiring intellectual property protection. In this latter case, it is recommended to first implement code with extensive functionality in an R package and license it appropriately, separately from the Shiny app. Data licensing generally falls under another type of licensing. The Creative Commons (or “CC”) licensing framework [9] is almost universally used. As with code, one of the permissive licenses, such as CC0 (no restrictions) or CC-BY (attribution required), is recommended as it will maximize potential data reuse. Most data repositories will suggest a default license for your deposit. Conversely, projects reusing data obtained elsewhere must comply with any licensing restrictions attached to the original data.

### 8. Make your data and source code citable

In addition to licensing your work, you can facilitate citation of your work and let people know about your expectations by providing instructions on how to cite your app code and data. One standard practice is to, in your repository’s README file, include the text “Cite this work as…” followed by a BibTeX [10] entry that users can cut and paste into their reference management tool. Another is to include a citation file, named CITATION.cff [11], in your project’s top-level directory. Versioning is very helpful when used in conjunction with citation because it allows users to refer to the exact version of the code or data they used. For your code, most code repository hosting providers have features enabling the creation of named versions or “releases.” Most of the data repositories also provide a versioning system and mechanisms to cite a specific version of your data, generally via issuing a DOI that can be added to the Shiny app UI (see companion app example).

### 9. Share your app

Of course, you will want to make your app available to users! Certainly, the easiest way to publish your app is by pushing it to shinyapps.io, a hosting platform provided by Posit, the maker of several analytical tools such as RStudio. The principal limitation of shinyapps.io is that it is a proprietary system run as a matter of courtesy by Posit. While Posit is a registered Public Benefit Corporation (PBC) whose mission is to support data science, it is nevertheless subject to marketplace pressures and the need for some service profitability. Furthermore, in addition to caps on application size, the limit on the number of minutes your app can run per month will preclude its being usable in all but the smallest cases. As soon as your app gets visited by more than a few people a month, a paid subscription plan will be required to keep it running. Finally, in terms of storage, the app/data bundle is limited to 1 GB as of this writing. Note that data stored on shinyapps.io is ephemeral (it disappears every time the app is launched), and therefore any data collected by the app that is intended to be permanent will have to be stored externally.

Another option to share your app is to use **shinylive** [12], which builds on WebAssembly via webR and thereby removes the need for a hosted Shiny server. This enables running Shiny apps entirely within a user’s browser. Essentially, the **shinylive** R package exports your Shiny application files to a directory that can be hosted on a static web server, such as GitHub Pages. Unfortunately, as of the writing of this manuscript, our testing with an R shiny app that loads a ~30 MB dataset and that uses only the **shiny** and **tidyverse** packages showed that **shinylive** was not stable nor fast enough to be usable and was inconsistent across machine configurations (OS and web browser used). However, as this approach gets further refined and more Shiny features are supported, it is time well spent to try it for your app due to the ease of maintenance provided by removing the need to rely on a dedicated Shiny server host.

Independent of publishing your app, you will also want to make your app available using a collaborative platform such as GitHub, as well as in an archival platform such as Zenodo. Hosted on GitHub and/or Zenodo, a Shiny app can be downloaded and run locally within RStudio or even directly from R [13]. In this way, a Shiny app can be viewed as a specialized file format that is openable by R. For additional convenience, the **shiny** package provides the runUrl() and runGitHub() functions that let you run an app directly from a download URL or GitHub repository, respectively. These functions automatically download the app files, unzip them in a temporary folder, and launch the app locally.

Other than perhaps running your own Shiny server (an option that generally requires a dedicated IT team to support), the above options are exhaustive as of this writing. The commonly used platforms for hosting Python and R notebooks (Google Colab, Binder, etc.), regrettably, do not support Shiny.

### 10. Consider lighter-weight alternatives

Shiny is undoubtedly a powerful tool for creating interactive applications that enable the development of powerful web applications. However, Shiny might not be the most efficient solution for you as it requires an upfront investment to develop the R/Python code and has substantial infrastructure and maintenance costs to run and maintain the code and the dedicated server necessary to run the custom underlying R/Python code. In fact, the first question of a Shiny project should be: Do you need to use Shiny at all? There are several other “ready-to-use” tools, often referred to as “HTML widgets,” that support interactively exploring data and that may meet your needs without requiring any infrastructure beyond ubiquitous web technologies. These tools cannot perform computation or take in user data, but they can provide basic data exploration, such as querying and filtering. Here are a few selected examples worth considering:

- Plotly [14] lets you build interactive plots that support customizable, pop-up tooltips, the ability to zoom in/out, an interactive legend to select categories, and more. It is integrated with **ggplot2** [15], making it trivial to convert a static **ggplot2** plot into a **Plotly** plot.
- DataTable [16] creates interactive tables, giving users the ability to sort columns and search for values.
- Leaflet [17] can be used to visualize and explore spatial data. It creates interactive, pannable, and zoomable maps and provides standard background maps.

Note that these HTML widgets can also be integrated into Quarto [18] documents, such as websites and dashboards [19].

## Conclusion

Shiny is a robust framework enabling R and Python communities to develop interactive web applications without needing to learn a new programming language. It is a fast-evolving ecosystem with new tools and packages been launched regularly, such as **shinylive** [20], **promises** [21], **bslib** [22], and AI-powered Shiny Assistant [23]. As a result, it has never been easier to develop powerful Shiny applications. Shiny complements scientific publications and engages stakeholders by allowing them to interact directly with the data underlying interactive data visualizations. We hope these 10 tips will help Shiny application developers to build more robust and easy-to-maintain Shiny applications and foster a mindset of reproducibility within the Shiny community that enables the reuse and repurposing of the many applications developed using this framework.

## Supporting information

S1 Text Code comments. Example for the User Interface (UI) code.
(PDF)

S2 Text Code to cache data locally. Example caching data from Zenodo.
(PDF)
