# Supplementary material for: Ten quick tips for developing a reproducible Shiny application
Source: PLoS Comput Biol. 2025 Oct 13;21(10):e1013551. doi: 10.1371/journal.pcbi.1013551 (PMC12517473; doi:10.1371/journal.pcbi.1013551)
Supplement: S1 Text — Example for the User Interface (UI) code. (PDF) [file pcbi.1013551.s001.pdf]

S1 Text. Code comments. Example for the User Interface (UI) code

```
navbarPage(
    …
    tabPanel(              # main tab
        fluidRow(          # top row
          ... other UI elements
        ),                 # end of top row
    ),                     # end of main tab
    ... other tabs
)
```
