# Supplementary material for: Ten quick tips for developing a reproducible Shiny application
Source: PLoS Comput Biol. 2025 Oct 13;21(10):e1013551. doi: 10.1371/journal.pcbi.1013551 (PMC12517473; doi:10.1371/journal.pcbi.1013551)
Supplement: S2 Text — Example caching data from Zenodo. (PDF) [file pcbi.1013551.s002.pdf]

S2 Text. Code to cache data locally. Example caching data from Zenodo

```
# Local filename
cache_file <- "data/app_data.csv"

# URL to remote file (here pretending to be on Zenodo)
remote_file <- "https://zenodo.org/records/4994321/files/app_data.csv"

# Load the remote data if necessary
if (!file.exists(cache_file)) {
    download.file(remote_file, cache_file)
}

# Always read from the local file
app_data <- read.csv(cache_file)
```
